# Nemo-like kinase regulates the expression of vascular endothelial growth factor (VEGF) in alveolar epithelial cells

**DOI:** 10.1038/srep23987

**Published:** 2016-04-01

**Authors:** Hengning Ke, Katarzyna Chmielarska Masoumi, Kristofer Ahlqvist, Michael J. Seckl, Kristina Rydell-Törmänen, Ramin Massoumi

**Affiliations:** 1 Molecular Tumor Pathology, Department of Laboratory Medicine, Lund University, Sweden; 2 Department of Medical Oncology, Imperial College Healthcare NHS Trust and Imperial College London, London, UK; 3 Lung Biology, Department of Experimental Medical Science, Lund University, Sweden

## Abstract

The canonical Wnt signaling can be silenced either through β-catenin-mediated ubiquitination and degradation or through phosphorylation of Tcf and Lef by nemo-like kinase (NLK). In the present study, we generated NLK deficient animals and found that these mice become cyanotic shortly before death because of lung maturation defects. NLK−/− lungs exhibited smaller and compressed alveoli and the mesenchyme remained thick and hyperplastic. This phenotype was caused by epithelial activation of vascular endothelial growth factor (VEGF) via recruitment of Lef1 to the promoter of VEGF. Elevated expression of VEGF and activation of the VEGF receptor through phosphorylation promoted an increase in the proliferation rate of epithelial and endothelial cells. In summary, our study identifies NLK as a novel signaling molecule for proper lung development through the interconnection between epithelial and endothelial cells during lung morphogenesis.

Lung development is a complex process requiring interplay between different cell types, which communicate through specific and regulated signaling pathways^1^. Inappropriate lung development causes diverse lung diseases including cystic fibrosis and asthma, and may contribute to chronic obstructive pulmonary disease (COPD)^2^. Similar to humans, lung development in mice is regulated by growth factors expressed by the epithelium or stromal cells, which in turn activate receptors in a paracrine or autocrine manner. Gene transcription mediated by receptor activation regulates the growth and differentiation of all compartments of the lung^3^,^4^. Wnt signaling is one of the most important pathways orchestrating normal lung development in mice and humans^5^.

The Wnt pathway is involved in different cellular process, such as cell fate decisions, stem cell self-renewal, cell proliferation, and differentiation. The importance of Wnt signaling was demonstrated by mutations in constituent steps, which cause developmental defects in mice^6^. In response to canonical Wnt binding to its receptors, β-catenin translocates from the cytosol to the nucleus, binds directly to the transcription factors T-cell factor (Tcf) and lymphoid enhancing factor (Lef), and activates transcription of Wnt target genes such as c-Myc and cyclin D1^7^,^8^. β-catenin, Tcf1-4, and Lef1 are all expressed in different lung cell types including the primordial epithelium (PE), alveolar epithelium (AE), and adjacent mesenchymal cells^9^. Mice harboring tissue-specific deletion of β-catenin in lung epithelial cells die at birth because of respiratory failure. This phenotype has been explained by disrupted lung morphogenesis, lack of differentiation of the peripheral lung, and enhanced formation of the conducting airways^10^. Lung-endoderm-specific expression of constitutively active β-catenin-Lef1, promotes uncontrolled proliferation and a lack of differentiation in lung cells^11^. Furthermore, deletion of Wnt5a causes hyperthickening of the mesenchymal interstitium and overbranching of the epithelial airway^12^, whereas overexpression of Wnt5a in the lung epithelium disrupts epithelial-mesenchymal interaction and causes malformations in both the airway epithelium and the surrounding mesenchyme^13^.

Nemo-like kinase (NLK) is a kinase activated by phosphorylation^14^ and is involved in differentiation and embryogenesis in *C. elegans, Xenopus*, and *Drosophila*^15^,^16^,^17^,^18^. Previous studies have demonstrated a tumor suppressor function of NLK, through regulating proliferation and cell survival of tumor cells originating from the breast, liver, gallbladder, and colon^19^,^20^,^21^,^22^. Upon activation, NLK can phosphorylate several proteins essential for the regulation of IL-6, Notch, and canonical Wnt/β-catenin signaling^15^,^16^,^23^,^24^,^25^,^26^. The negative regulation of Wnt signaling by NLK is mediated via the phosphorylation of Tcf/Lef. Phosphorylation of this complex by NLK prevents the recruitment of Tcf/Lef to DNA and facilitates ubiquitination and degradation of the complex^15^.

In the present study, we generated NLK deficient mice and found that these mice had a severe phenotype with a very short lifespan, i.e. they die within 12–36 hours after birth. The reason for this phenotype was found to be an abnormal pulmonary architecture, with a skewed balance between endothelial and epithelial development. More precisely, CD31-positive endothelial cells showed elevated proliferation and hyperplasia caused by alveolar epithelial cell-dependent aberrant expression of VEGF.

## Results

**Figure 1:**
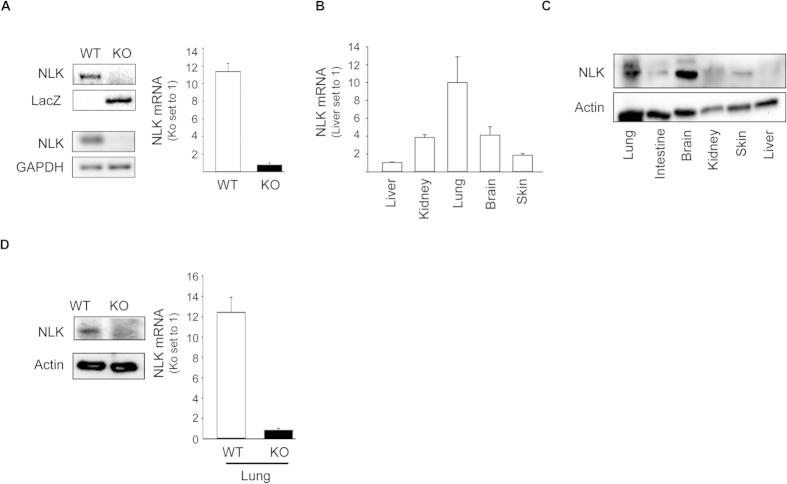
Generation of NLK deficient animals. **(A)** The insertion of the stop cassette was shown by genotyping analysis of wild type and NLK knockout mice (Left top panel). RT and qRT-PCR of total RNA from mouse embryonic fibroblast isolated from E 13.5 wild type and NLK knockout mice. GADPH was used as a loading control. **(B,C)** Protein and mRNA expression levels of NLK in wild type mouse tissues including lung, brain, skin, kidney, and liver. The blot in figure (**C**) has been cut in two pieces and blotted against NLK or actin under the same experimental conditions. **(D)** Protein and mRNA expression levels of NLK expressed in lung tissues at birth (P1) of wild type and NLK knockout mice. The blot in this figure has been cut in two pieces and blotted against NLK or actin under the same experimental conditions.

To explore the function of NLK *in vivo*, we generated NLK-deficient mice by inserting a lacZ reporter gene upstream of the exon 2 of NLK gene (Supplementary Fig. S1). We confirmed the absence of NLK mRNA and protein in mouse embryonic fibroblasts by Western blot and RT-PCR analyses (Fig. 1A). In the lung, brain, and kidney tissues of new born mice, NLK showed the highest expression compared to other tissues tested (Fig. 1B,C). Immunohistochemistry, Western blot, and RT-PCR analyses confirmed the absence of NLK expression in the lungs and brains of NLK deficient mice (Fig. 1D and Supplementary Fig. S2A,B). Immunohistochemical analysis also provided evidence that NLK is mainly localized in the nucleus of cells isolated from lung or brain tissues in wild type animals using NLK antibodies (Supplementary Fig. S2A,B), whereas β-gal expression was only detected in the lung and brain tissues isolated from NLK knockout animals (Supplementary Fig. S2A,B).

**Figure 2:**
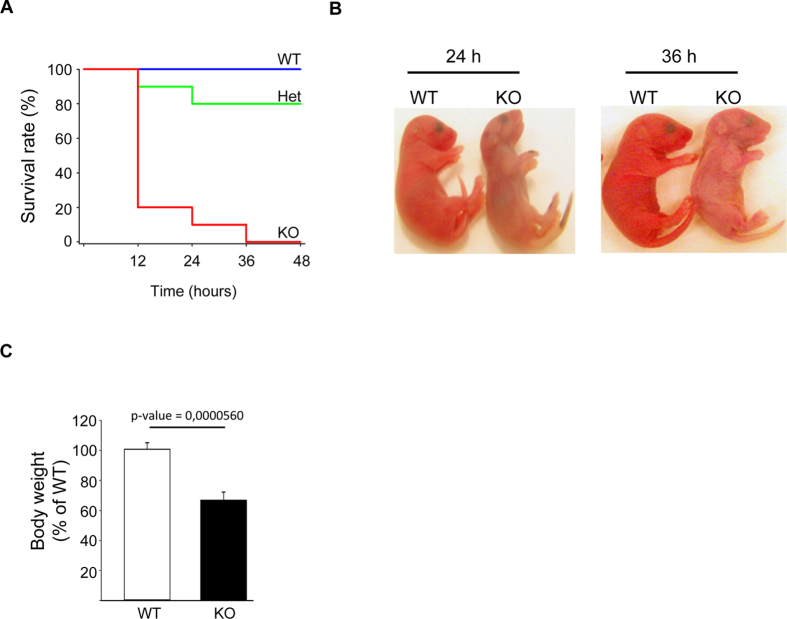
NLK deficient animals have a severe phenotype with a short lifespan. **(A)** The survival rate of wild type (n = 74), heterozygous (n = 123) and NLK knockout mice (n = 59) between postnatal day 1 and day 3. (**B**) Images of NLK+/+ (WT) and NLK−/− (KO) mouse 24 hours (left panel) and 36 hours (right panel) after birth. (**C**) Relative birth weight of wild type NLK and NLK−/− pups.

NLK deficient animals had a severe phenotype with a short lifespan. All of the NLK deficient mice were alive at birth but they died within 12–36 hours after birth (Fig. 2A,B). Most of the heterozygous mice had no obvious phenotype (Fig. 2A). Examination of newborn NLK−/− mice showed that these animals were smaller than normal: average birth weight was only 72% of the average birth weight of their control littermates (Fig. 2C). Although their lungs inflated well with air at birth (Supplementary Fig. S3), many NLK−/− mice became cyanotic shortly before death (Fig. 2B, right panel). In our system, we took the advantage of FRT sequence around the introduced stop codon to ensure that the observed phenotype was dependent on NLK deletion (Supplementary Fig. S1). Crossing heterozygous NLK mice with FRT mice resulted in the removal of the stop codon from knockout animals which rescued the expression of NLK. These mice (FRTNLK) were healthy and could survive for up to one year without any obvious phenotype.

**Figure 3:**
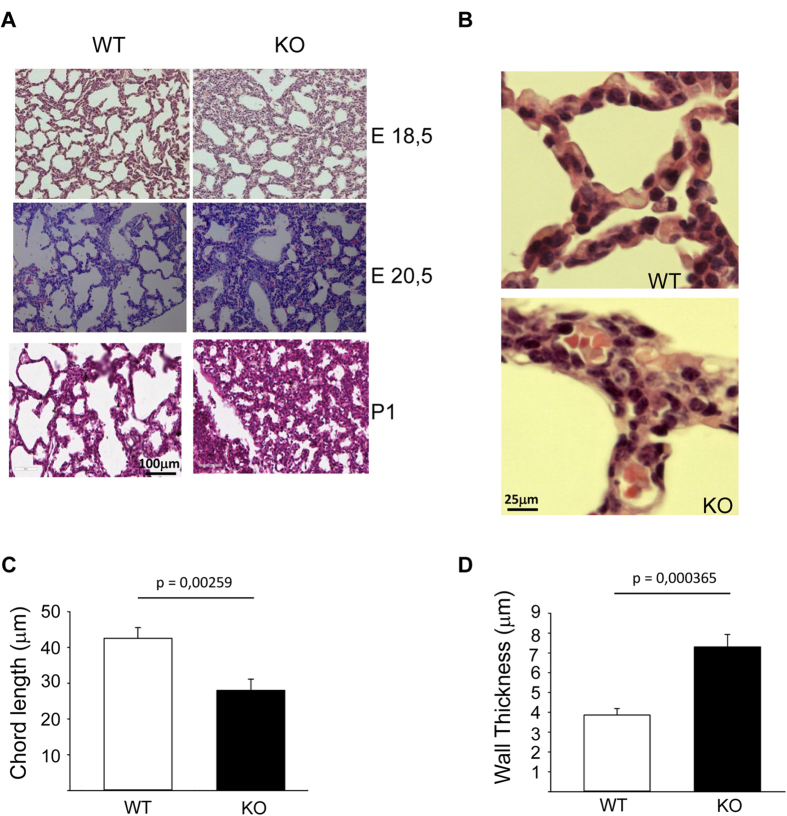
Smaller alveoli with irregular morphology and hyperplastic in NLK−/− compared to wild type mouse. **(A)** H&E-stained lung sections from E18.5, E 20.5, and P1 isolated form NLK+/+ and NLK−/− mice. **(B)** H&E-stained lung sections show the different appearance of alveolar septum between NLK+/+ and NLK−/− mice at P1. **(C)** The mean chord length of alveoli in NLK+/+ and NLK−/− mice at P1. **(D)** The thickness of the alveolar capillary wall in NLK+/+ and NLK−/− mice at P1.

Compromised lung development might be a contributing factor to the lethal cyanotic phenotype of NLK−/− mice. To test this hypothesis, we performed a histological analysis of NLK+/+ and NLK−/− mouse lungs (P1) to determine the impact of the ablation of NLK on lung architecture. By P1, NLK+/+ mouse lungs showed well-inflated alveoli and the mesenchyme had thinned out. However, NLK−/− lungs exhibited smaller and compressed alveoli and the mesenchyme remained thick (Supplementary Fig. S4). Higher magnification images showed a disorganized alveolar structure and thick alveolar walls (Supplementary Fig. S4). Next, we analyzed lung tissues isolated from embryos at E18.5. Much smaller alveoli with irregular morphology in knockout animals compared to NLK wild type mice were observed at E18.5, E20.5, and P1 (Fig. 3A,B). Quantification showed a reduction in the chord length (Fig. 3C) and increased thickness of the alveolar capillaries (Fig. 3D) in NLK−/− mice compared to wild type animals.

**Figure 4:**
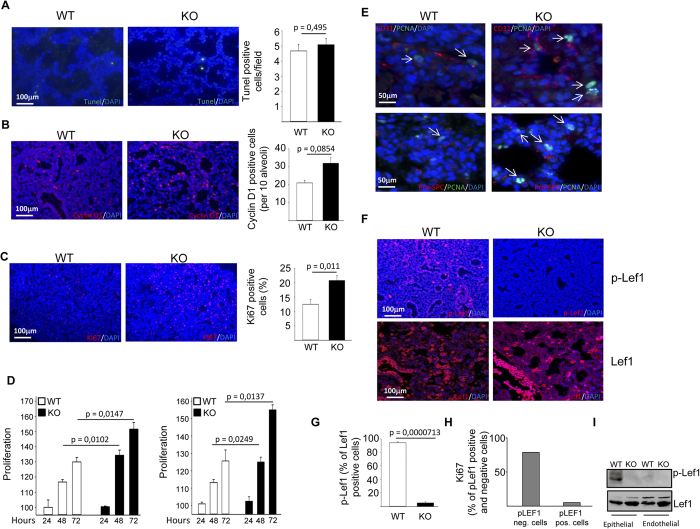
Increased cell proliferation in NLK−/− lung sections compared to wild type animals. **(A)** TUNEL staining and quantification of the number of positive cells in NLK+/+ and NLK−/− lung tissues at P1. **(B)** Cyclin D1 staining and quantification of the number of positive cells in NLK+/+ and NLK−/− lung tissues at P1. **(C)** Ki67 staining and quantification of the number of positive cells in NLK+/+ and NLK−/− lung tissues at P1. **(D)** Measurement of the growth rate of NLK+/+ and NLK−/− primary murine alveolar epithelial (left panel) and endothelial cells (right panel) over a period of 24–72 hours using MTS assay. **(E)** Double staining of PCNA and CD31 or PCNA and proSP-C in NLK+/+ and NLK−/− lung tissues at P1. **(F)** Immunofluorescence staining of total Lef1 and phospho-Lef1 positive cells in the lung sections isolated from NLK+/+ and NLK−/− at P1. **(G)** Quantification of the number of phospho-Lef1 positive cells among Lef1 positive cells in the lung sections isolated from NLK+/+ and NLK−/− at P1. **(H)** Quantification of the number of Ki67 positive cells among phospho-Lef1 positive cells in the lung sections isolated from NLK+/+ at P1. **(I)** The levels of total Lef1 and phospho-Lef1 in NLK+/+ and NLK−/− primary murine alveolar epithelial and endothelial cells. The blot in this figure has been blotted against pLef1. The membrane has been stripped and used again for blotting against total Lef1 under the same experimental conditions.

The phenotype of lungs from NLK deficient mice could be due to decreased apoptosis and/or increased proliferation. To determine the extent of apoptosis in NLK+/+ and NLK−/− lungs, we performed TUNEL staining of E18.5 lung tissues. This experiment revealed that the loss of NLK did not grossly affect cell survival (Fig. 4A). To examine cell proliferation, we compared BrdU, Ki67 and cyclin D1 expression between NLK+/+ and NLK−/− lung sections isolated from E18.5 embryos. NLK−/− lungs displayed a significant increase in all three proliferation markers compared to lung sections from wild type animals (Fig. 4B,C and Supplementary Fig. S5). To distinguish cell type specificity, we investigated proliferation status of pulmonary epithelial and endothelial cells. Higher number of proliferating alveolar epithelial type II (Fig. 4D, left panel) and endothelial cells (Fig. 4D, right panel) isolated from knockout mice compared to wild type animals could be observed. This result was confirmed using lung sections isolated from E18.5 embryos and stained for pro-surfactant protein C (proSP-C) positive alveolar epithelial type II and endothelial cells (Fig. 4E and (Supplementary Fig. S6). We could not observe any significant differences in the proliferation of aquaporin-5 (AP-5) positive alveolar epithelial type I cells comparing wildtype with NLK deficient mice (Supplementary Fig. S6). To analyze whether NLK regulates Wnt-mediated proliferation through the phosphorylation of Lef1, we performed immunofluorescent staining of lung tissues using phosho-Lef1 antibodies. No differences in the total number of Lef1 positive cells could be observed, whereas the number of phospho-Lef1 positive cells was three times higher in wild type mice compared to NLK deficient lung tissues (Fig. 4F,G). In wildtype tissue we found a negative correlation between cells positive for proliferation marker (Ki67) and Lef1 phosphorylation (Fig. 4H). Furthermore, wildtype isolated pulmonary epithelial but not endothelial cells showed higher levels of Lef1 phosphorylation compared with NLK deficient cells (Fig. 4I). Taken together, these results suggest that deletion of NLK promotes proliferation of pulmonary cells through the Lef signaling pathway operating in epithelial cells.

**Figure 5:**
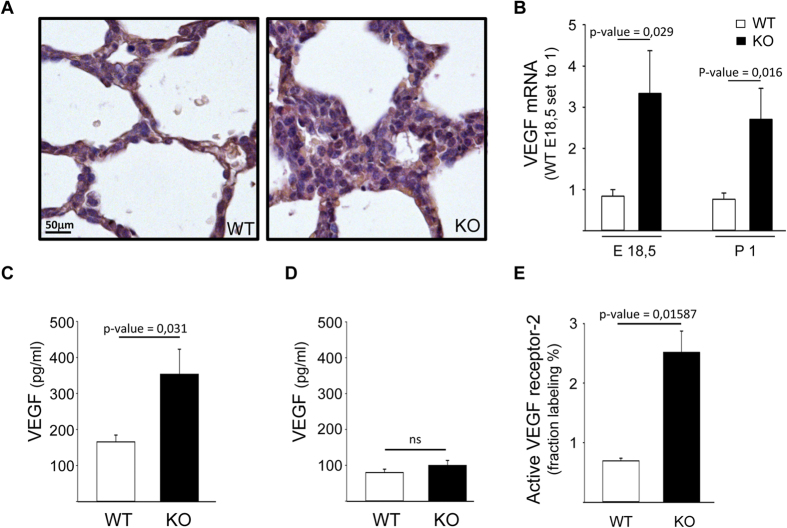
Hyperthickening of the lung vascularization and VEGF-A expression. **(A)** H&E-stained lung sections from showing hyperthickening of the lung vascularization in lung sections from NLK+/+ and NLK−/−. **(B)** The levels of VEGF-A at the mRNA levels in NLK+/+ and NLK−/− lung tissues isolated from E18.5 and P1**. (C)** The levels of secreted VEGF in the culture media of NLK+/+ and NLK−/− primary epithelial cells. **(D)** The levels of secreted VEGF in the culture media of NLK+/+ and NLK−/− primary pulmonary endothelial cells. **(E)** Immunofluorescence staining and quantification of phosphorylated VEGF receptor 2 (VEGFR2) in lung sections isolated from NLK+/+ and NLK−/− at P1. Heat-mediated antigen retrieval in high pH buffer was performed before labeling. Data is given as fraction labeling (%); percentage positively labeled area of the total lung tissue area.

In addition to the hyperthickening of the lung vasculature in knockout compared to wild type tissues (Fig. 5A) lung tissues isolated from wild type and NLK deficient animals, showed a significant differences in the levels of vascular endothelial growth factor (VEGF) mRNA at E18.5 and P1 (Fig. 5B). VEGF isoform 188 compared to 120 and 164 was highly expressed in NLK−/− lung sections isolated from P1 (Supplementary Fig. S7). This is in agreement with previous study showing that VEGF188 is highly expressed in alveolar cells in the mouse during development^27^. Epithelial cells isolated from knockout animals secreted higher levels of VEGF-A compared to wild type cells (Fig. 5C). Endothelial cells also secreted VEGF-A, but at lower levels compared to epithelial and no differences in VEGF expression could be observed comparing wild type and NLK knockout cells (Fig. 5D). Furthermore, immunofluorescent staining using antibodies recognizing the active form of VEGF receptor-1 (VEGFR-1) or VEGFR-2 showed increased numbers of cells harboring phosphorylated VEGFR-2 (Fig. 5E) in both epithelial and endothelial NLK deficient cells (Supplementary Fig. S8). The staining of VEGFR1 phosphorylation was weak (Supplementary Fig. S9), suggesting that VEGFR-2 activation is the major VEGF receptor mediating downstream signaling.

**Figure 6:**
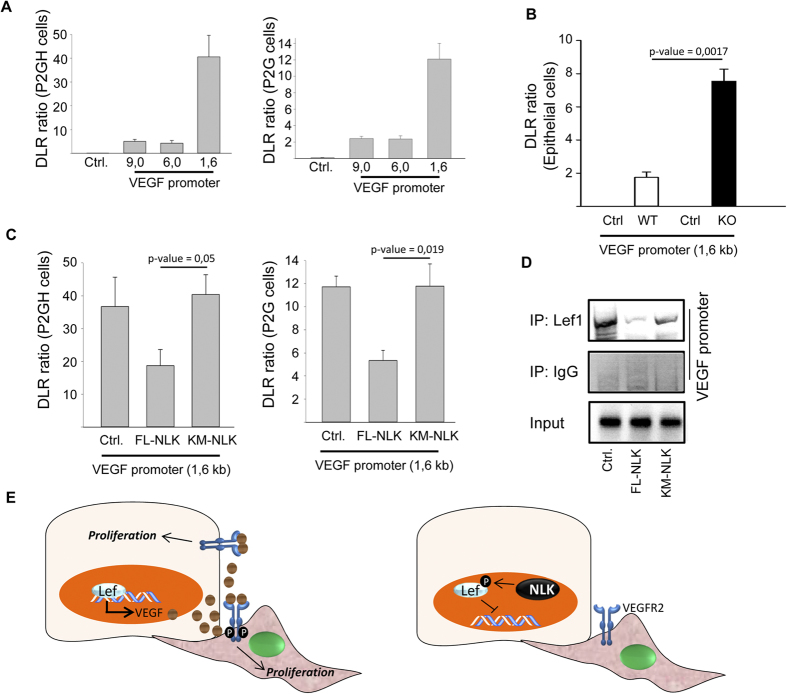
Recruitment of Lef1 to the promoter of VEGF-A. **(A)** Luciferase activity in human lung epithelial cells P2GH (left panel) and P2G (right panel) using deletion mutants of VEGF promoter. Control (Ctrl): cells without transfection with luciferase expression plasmid. **(B)** VEGF promoter luciferase activity (1,6 kb) in primary isolated mouse lung epithelial cells. Control (Ctrl): cells without transfection with luciferase expression plasmid. **(C)** Luciferase activity in human lung epithelial cells P2GH (left panel) and P2G (right panel) using deletion mutant of VEGF promoter (1,6 kB) in cells transfected with Full length (FL-NLK), catalytically inactive mutant of NLK (KM-NLK), or empty expression vector (Ctrl). **(D)** Recruitment of Lef1 to the VEGF-A promoter by ChIP using human lung epithelial cells (P2GH) transfected with Full length (FL-NLK), catalytically inactive mutant of NLK (KM-NLK), or empty expression vector (Ctrl). IP: Lef1 indicates immunoprecipitation (IP) using polyclonal antibodies. IP: IgG, IP using pre-immune serum. Input: 10% of the cell lysate used for the IP is shown. **(E)** Model of how deletion of NLK leads to elevated levels of VEGF expression causing aberrant proliferation of pulmonary epithelial and endothelial cells (left). In the wildtype epithelial cells, NLK mediated-phosphorylation of Lef1 prevents the binding of Lef1 to the promoter of VEGF (right).

To determine the transcriptional elements necessary for VEGF-A upregulation, we overexpressed deletion mutants of the VEGF-A promoter using a luciferase reporter or the promoterless construct (pGL2-basic) containing only the firefly luciferase-reporter in two different immortalized human alveolar cell lines, i.e. P2G and P2GH^28^,^29^. Luciferase activity was observed for all VEGF-A promoters tested, but the 1.6 kb deletion mutant showed the highest activity, suggesting that this region contains regulatory elements for VEGF-A expression (Fig. 6A). Indeed, epithelial cells isolated from knockout animals showed higher levels of VEGF-A promoter activity compared top wild type cells using 1.6 kb deletion mutant (Fig. 6B). In this region, 1394 bp before the start codon, we could identify Tcf binding element (TBE) (Supplementary Fig. S10; labeled in red). Overexpression of wild type NLK but not the catalytic mutant NLK (NLK-K155M) in P2G and P2GH cells reduced the promoter activity of VEGF-A, suggesting that activation of NLK is necessary for this effect (Fig. 6C). Furthermore, chromatin immunoprecipitation using epithelial cells showed that overexpression of NLK, but not NLK-K155M, reduced the binding of Lef1 to the VEGF-A promoter in human alveolar P2GH cells (Fig. 6D). These results suggest that expression of VEGF-A by epithelial cells is regulated by the activation of NLK.

## Discussion

Wnt receptor activation via β-catenin and Tcf/Lef is involved in different aspect of mouse and human tissue development^30^. Beside degradation of β-catenin via proteasome, this signaling pathway can be antagonized by NLK. In the present study, we investigated whether degradation of β-catenin via proteasome overrides the function of NLK for limitation of Wnt signaling. We found that deletion of NLK leads to shorter lifespan, where the pups succumb within 12–36 hours after birth, and this was caused by a respiratory phenotype causing breathing problems. The newborn NLK deficient mice were smaller than normal and became cyanotic shortly before death. Investigating tissue specific expression of NLK, we found the highest NLK levels in lungs and brain of new born mice. Recently, it was shown that generation of NLK-deficient mice provided a phenotype that varied with the genetic background^31^. In our system crossing heterozygous NLK mice with FRT mice resulted in removal of stop codon from the knockout animals and prolonged their survival to one year of age.

The phenotype of NLK deficient mice is caused by smaller and compressed alveoli as well as thick and edematous alveolar walls, which could be explained by an increase in cell proliferation. Previously it has been shown that NLK is negatively regulate Wnt/β-catenin signaling by phosphorylation of the complex Tcf/Lef and preventing DNA association of these transcription factors^23^. Since we found a negative correlation between proliferation and Lef1 phosphorylation in wild type mice, this suggests that, in the absence of NLK, inactivation and phosphorylation of Lef1 is disrupted. Non-phosphorylated Lef1, in a complex with β-catenin and Tcf, promotes the transcriptional activation of target genes including cyclin D1, which causes an increase in the proliferation rate of cells. Previous studies have demonstrated that low NLK expression is an independent prognostic factor for the survival of patients suffering from non-small cell lung cancer (NSCLC). Moreover, in cell culture, downregulation of NLK resulted in significantly increased proliferation in NSCLC cells through the transcriptional activity of the Wnt signaling pathway via β-catenin and Tcf/Lef activation^32^.

Aberrant expression of VEGF has been linked to several lung diseases, including acute lung injury, severe pulmonary hypertension, and emphysema^33^. Expression and synthesis of VEGF is controlled by several signaling pathways. VEGF and VEGFR are expressed during embryogenesis and VEGFR signaling plays an essential role in lung development^33^,^34^. The main receptors of VEGF-A is VEGFR-1 and VEGFR-2. Activation of VEGFR-2 has been shown to mediate mitogenic signal^35^, whereas VEGFR-1 activation is believed to act as a decoy receptor by inhibiting VEGF binding to VEGFR-2^36^,^37^,^38^. However, it has not yet been established whether lung alveolar epithelial cells can regulate VEGF-A through Wnt signaling, and furthermore which region in VEGF-A promoter recruits Lef1. We could identify part of the VEGF promoter, to which Lef1 was recruited and promoted an increase in the expression of VEGF. We could also show that the activation of NLK was necessary for repressing the activation of the VEGF promoter since overexpression of a catalytically inactive mutant of NLK in knockout cells was unable to prevent the association of Lef1 to the VEGF promoter or decreased VEGF promoter activity. Our results suggest that Wnt-induced NLK activation and subsequent Lef1 phosphorylation repress VEGF expression during embryogenesis; this pathway is pivotal for correct lung development. Previously it has been demonstrated that deletion of Wls (receptor protein directing Wnt ligands from the Golgi to the cell surface) from the embryonic pulmonary inhibits growth and differentiation of peripheral lung as well as formation of the pulmonary vasculature. In this pathway, epithelial Wls was required for appropriate expression of essential regulators of vascular development including VEGF-A^39^.

In the present study, we found that in the absence of NLK led to aberrant expression of VEGF via β-catenin-Tcf signaling in lung epithelial cells (Fig. 6E). The expression of VEGF-A promotes the proliferation of pulmonary cells and vascular hyperthickening. In NLK-deficient animals, the disrupted Wnt balance causes cellular hyperplasia and thickened alveolar walls during lung morphogenesis that together leading to limited survival of these animals.

## Methods

### Generation of knockout mice

*NLK* knockout (KO or *NLK*−/−) mice were generated by inserting a cassette containing lacZ reporter gene upstream of exon 2 of the NLK gene. This construct also contains a short loxP sequence inserted up- and downstream of exon 2, creating a floxed gene (Supplementary Fig. 1). In addition, this construct contains FRT sequences flanking the stop codon. Crossing of NLK+*/−* mice with mice bearing an introduced Flp gene led to the removal of the flanked cassette containing the splice receiver, thus redirecting the splicing to exon 2 and restore the expression of NLK in their NLKfl/fl (Supplementary Fig. S1). All mice were maintained in specific pathogen-free housing at the Clinical Research Centre in Malmö, and the animal experiments were performed according to the national and international guidelines of the European Union. Furthermore, all experimental protocol was approved by Swedish regional (Malmö-Lund) ethical committee (application number: M336-12).

Mice were weighted instantly after birth with a scale and observed for life span every two hours. Life time was recorded as the time between the death time point found and the birth time found.

Mice were genotyped using primers:

NLKForward 5′-GGGACTTCAGAGACAGGGAAC-3′

NLKReverse 5′-CTGGAAGACGTTGGGCATC-3′

LacZForward 5′-GCATCGAGCTGGGTAATAAGCGTT-3′

LacZReverse 5′-GACACCAGACCAACTGGTATTGG-3′

FRTForward 5′-AAA GTC GCT CTG AGT TGT TAT-3′

FRTReverse 5′-GCG AAG AGT TTG TCC TCA ACC-3′

### Human alveolar cells

Human alveolar type II pneumocytes were isolated from human lung resection specimens^29^ and immortalized by transduction with the catalytic subunit of telomerase and simian virus 40 large-tumor antigen as previously described^28^. The methods involving human tissue were carried out in accordance with the approved guidelines. Local institutional review board approval was given for the anonymous collection of lung tissue. Ethical approval was obtained from the Brompton, Harefield, and NHLI Ethics Committee, and patients gave informed consent.

### Isolation of primary murine alveolar epithelial, endothelial cells and ELISA assay

Isolation of primary murine type II alveolar epithelial cells^40^ and isolation and culture of pulmonary endothelial cells from neonatal mice^41^ were performed as previously described.

The analysis of mouse VEGF expressed in cell culture was performed by using VEGF Quantikine ELISA Kit (MMV00, R&D systems) according to manufactures descriptions.

### Determination of alveolar airspace size, number of primitive alveoli and number of alveolar septa

The size of the alveolar airspace was determined by measuring mean chord lengths on H&E-stained lung sections (Bry *et al.*, 2007). Images were taken from five representative non-overlapping fields of lungs from at least eight mice. A grid consisting of six lines at 35 μm intervals was overlaid on the image. Areas of bronchiolar airways and blood vessels were eliminated from the analysis. The length of the lines overlapping the alveoli was measured and averaged to give the mean chord length of the alveolar space. The number of primitive alveoli and secondary alveolar septa was determined on H&E-stained E18.5 and P1 lung sections, respectively. Primitive alveoli of E18.5 lungs were identified as pouches or sacs with flattened epithelium radiating from the terminal airways. Secondary septa on P1 lungs were identified as the thin membranes that folded into the alveolar sac.

### Chromatin immunoprecipitation

Chromatin immunoprecipitation was performed according to the manufacturer’s instructions (IMGENEX Corporation), using a rabbit polyclonal Lef1 antibody (Abcam). The extracted DNA was used for semi-quantitative PCR to amplify the promoter region of the VEGF gene harboring Tcf-Lef binding elements. The primer sequences used in real-time quantitative PCR were:

VEGF (prim8) Forward 5′-CTTTAGCCAGAGCCGGGGT-3′

VEGF (prim8) Reverse 5′-ACCAAGGTTCACAGCCTGAAA-3′.

### Statistical analyses

Statistical analyses were performed using GraphPad Prism5 Software (GraphPad Software, San Diego, CA, USA). Results are expressed as mean ± s.e.m. or as percentages. P-values < 0.05 were deemed statistically significant. Comparisons between groups were performed using the Mann-Whitney U test. A correlation analysis was performed by determining the Pearson product-moment correlation coefficient.

## Additional Information

**How to cite this article**: Ke, H. *et al.* Nemo-like kinase regulates the expression of vascular endothelial growth factor (VEGF) in alveolar epithelial cells. *Sci. Rep.* **6**, 23987; doi: 10.1038/srep23987 (2016).

## Supplementary Material

Supplementary Information
