# Supplementary material for: Nemo-like kinase regulates the expression of vascular endothelial growth factor (VEGF) in alveolar epithelial cells
Source: Sci Rep. 2016 Apr 1;6:23987. doi: 10.1038/srep23987 (PMC4817507; doi:10.1038/srep23987)
Supplement: Supplementary Information [file srep23987-s1.pdf]

Nemo-like kinase regulates the expression of vascular endothelial growth factor (VEGF) in alveolar epithelial cells

Hengning Ke, Katarzyna Chmielarska Masoumi, Kristofer Ahlqvist, Michael J. Seckl, Kristina Rydell-Törmänen, Ramin Massoumi

Supplementary Fig. 1

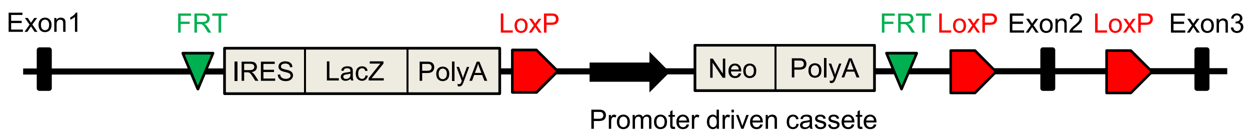

Supplementary Fig. 2

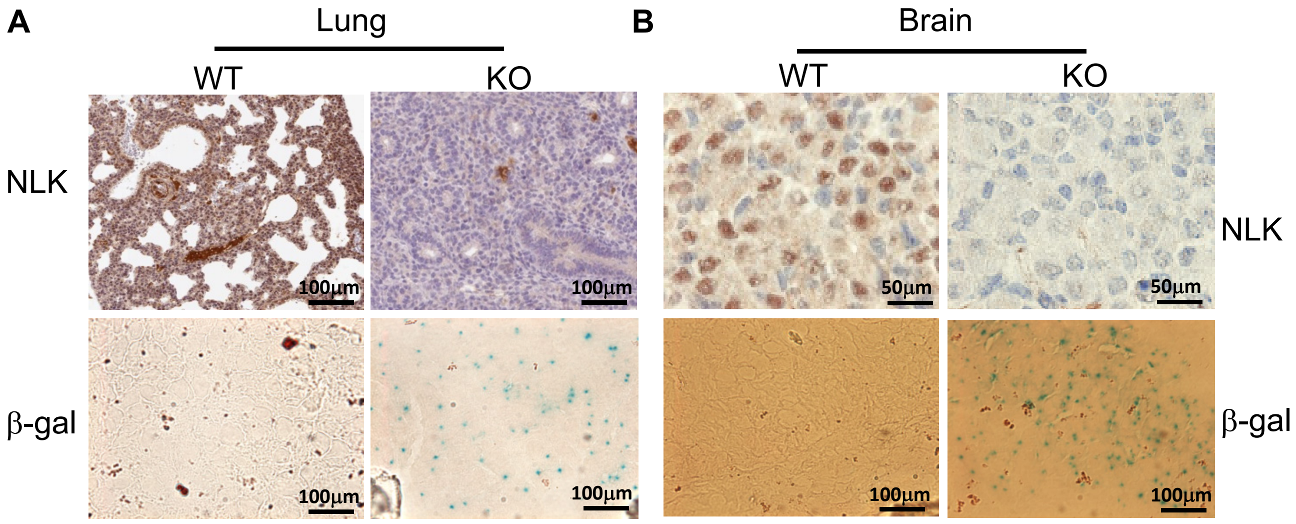

Supplementary Fig. 1
Generation of NLK deficient animals
(A) *NLK* deficient animals were generated by inserting a lacZ reporter gene upstream of exon 2 of the NLK gene. This construct also contains FRT sequences around the stop codon and short loxP sequences inserted up- and downstream of the targeted exon 2 creating a floxed gene. Crossing NLK mice with a FRT strain mouse will lead to removal of the stop codon and expression of NLK.

Supplementary Fig. 2
NLK is highly expressed in mouse lung tissue
(A-B) Immunohistochemistry and histological analysis of NLK using anti-NLK antibodies, and LacZ expression using beta-galactosidase staining kit, in the lungs and brain of wild type and NLK knockout mice.

Supplementary Fig. 3

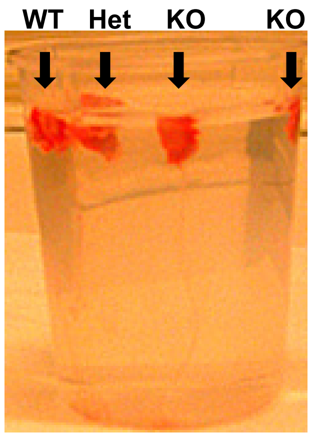

Supplementary Fig. 3
NLK deficient mouse lungs inflated well with air at birth
The floating lung assay for NLK+/+, NLK+/-, and NLK-/- newborns indicating that lungs inflate well with air at birth.

Supplementary Fig. 4

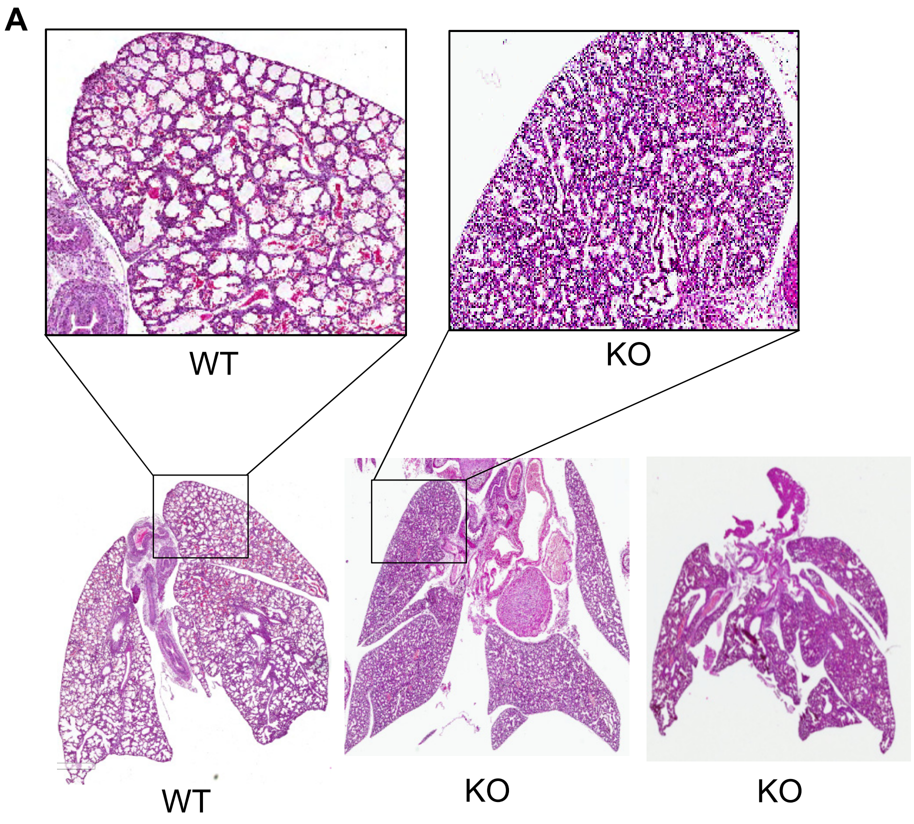

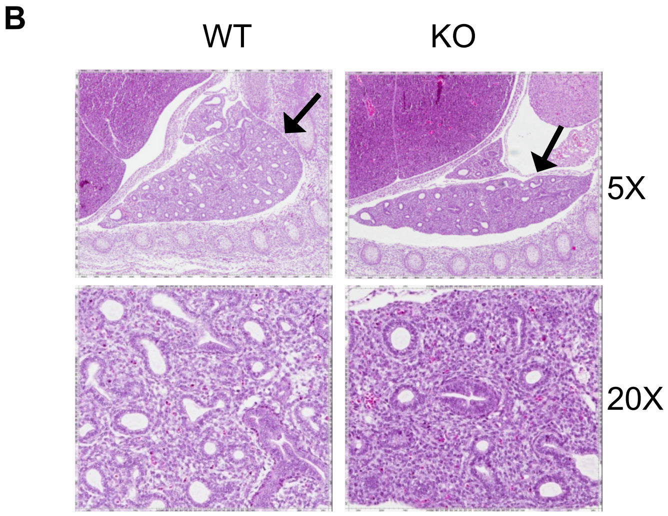

Supplementary Fig. 4
Smaller alveoli in NLK-/- compared to wild type mouse
(A) Frontal sections of lungs and enlarged sections from NLK+/+ and two NLK-/- mice at birth.
(B) Parasagittal sectioning of lungs (arrows) from NLK+/+ and NLK-/- mice at E15.5.

Supplementary Fig. 5

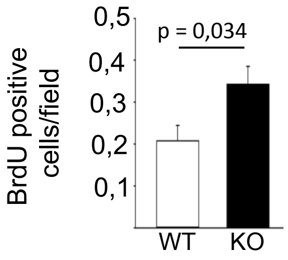

Supplementary Fig. 5
BrdU pulsing, staining, and quantification of the number of positive cells in NLK+/+ and NLK-/- lung tissues.

Supplementary Fig. 6

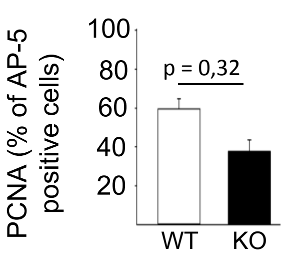

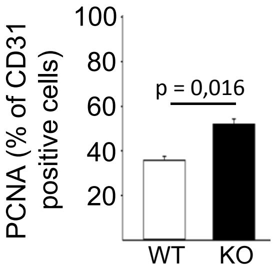

Supplementary Fig. 6
Double staining of PCNA and AP5 or CD31, and quantification of the number of positive cells in NLK+/+ and NLK-/- lung tissues.

Supplementary Fig. 7

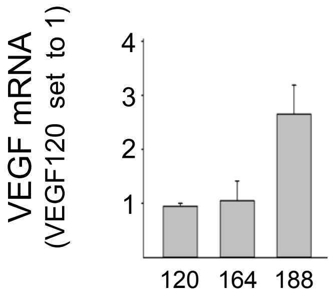

Supplementary Fig. 7
The levels of VEGF120, VEGF164, and VEGF188 at the mRNA levels in NLK-/- lung tissues isolated from P1.

Supplementary Fig. 8

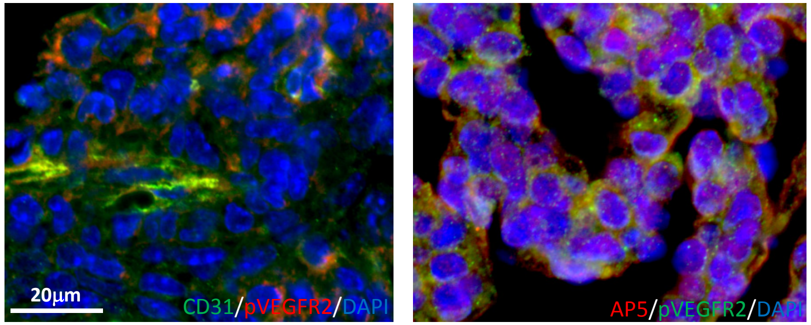

Supplementary Fig. 8
Immunofluorescence staining of phosphorylated VEGF receptor 2 (pVEGFR2) in lung sections isolated from NLK-/- at P1.
Left image: green: CD31, red: Phospho-VEGF receptor 2, blue: DAPI
Right image: green: Phospho-VEGF receptor 2, red: AP5, blue: DAPI

Supplementary Fig. 9

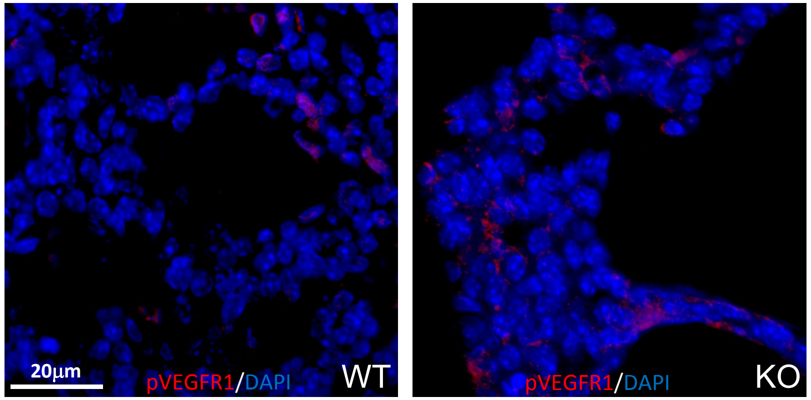

Supplementary Fig. 9
Immunofluorescence staining of phosphorylated VEGF receptor 1 (pVEGFR1) in lung sections isolated from NLK+/+ and NLK-/- at P1.
Red: Phospho-VEGF receptor 1
Blue: DAPI

Supplementary Fig. 10

TBE

ccggctctccgccttcccctgcccccc**ttcaat**attcctagcaaagagggaacggctctcaggccctgtccgcacgtaacctcactttcc
tgctccctcctcgccaatgccccgcgggcgcgtgtctctggacagagtttccgggggcggatgggtaattttcaggctgtgaaccttg
gtgggggtcgagcttccccttcattgcggcgggctgcgggccaggcttcactgggcgtccgcagagcccgggcccgagccgcgtgtg
gaggggctgaggctcgcctgtccccgcccccggggcgggccgggggcggggtcccggcggggcggagccatgcgcccccccttt
ttttttaaaagtcggctggtagcggggaggatcgcggaggcttggggcagccgggtagctcggaggtcgtggcgctgggggctag
caccagcgctctgtcgggaggcgcagcggttaggtggaccggtcagcggactcaccggccagggcgctcggtgctggaatttgata
ttcattgatccgggttttatccctcttctttttcttaaacattttttttaaaactgtattgtttctcgttttaatttatttttgcttgccattcc
ccacttgaatcgggccgacggcttggggagattgctctacttccccaaatcactgtggattttggaaaccagcagaaagaggaaaga
ggtagcaagagctccagagagaagtcgaggaagagagagacggggtcagagagagcgcgcgggcgtgcgagcagcgaaagcg
acaggggcaaagtgagtgacctgcttttgggggtgaccgccggagcgcggcgtgagccctcccccttgggatcccgcagctgacca
gtcgcgctgacggacagacagacagacaccgcccccagccccagctaccacctcctccccggccggcggcggacagtggacgcgg
cggcgagccgcgggcaggggccggagcccgcgcccggaggcggggtggagggggtcggggctcgcggcgtcgcactgaaactttt
cgtccaacttctgggctgttctcgcttcggaggagccgtggtccgcgcgggggaagccgagccgagcggagccgcgagaagtgcta
gctcgggccgggaggagccgcagccggaggagggggaggaggaagaagagaaggaagaggagagggggccgcagtggcgact
cggcgctcggaagccgggctcatggacgggtgaggcggcggtgtgcgcagacagtgctccagccgcgcgcgctccccaggccctg
gcccgggcctcgggccggggaggaagagtagctcgccgaggcgccgaggagagcgggccgccccacagcccgagccggagagg
gagcgcgagccgcgccggccccggtcgggcctccgaaacc**atg**aactttctgctgtcttgggtgcattggagccttgccttgctgctc
tacctccaccatgccaaggtaagcggtcgtgc

Supplementary Fig. 10
Location of Tcf Binding Element (TBE, labeled in red) at VEGF-A promoter. atg (underline) shows start of translation as the first in-frame ATG codon.

Nemo-like kinase regulates the expression of vascular endothelial growth factor (VEGF) in alveolar epithelial cells

Hengning Ke, Katarzyna Chmielarska Masoumi, Kristofer Ahlqvist, Michael J. Seckl, Kristina Rydell-Törmänen, Ramin Massoumi

Supplemental Material

Histopathology and immunohistochemistry

Immunohistochemical staining of 5-μm sections from paraffin-embedded blocks was performed using antibodies against NLK (rabbit polyclonal, 1:100, Abcam), cyclin D1 (rabbit polyclonal, 1:50, from Abcam), phospho-VEGFR1-pTyr1333 (rabbit polyclonal 1:100, from Sigma Aldrich), phospho-VEGFR2-pTyr1175 (rabbit polyclonal 1:100, from Cell signaling), total Lef1 (rabbit polyclonal, 1:100, Thermo Scientific), phospho-Lef1 (mouse monoclonal, 1:100, Millipore), pro-surfactant protein C (rabbit polyclonal, 1:500, Abcam), and Aquaporin 5 (rabbit polyclonal, 1:500, Abcam), using an indirect immunoperoxidase protocol according to the LSAB2-kit (DAKO, Hamburg, Germany). For the negative control, the primary antibody was omitted, and IgG isotype control antibodies did not reveal any detectable staining. Antibody binding was visualized using AEC solution (LSAB2-Kit, DAKO). For immunofluorescent detection, heat-mediated antigen retrieval was performed before application of the primary antibody, and for visualization tissue sections were incubated with AlexaFluor488 (green) or AlexaFluor548 (red) conjugated secondary antibodies, then counterstained with DAPI. Images were taken using an Olympus BX41 microscope.

Analysis of cell proliferation using BrdU

To analyze cell proliferation, mice were injected i.p. with a single dose of BrdU (3 μg/g of body weight; Roche) 2 hours prior to sacrifice. Lungs were isolated, fixed in 4% paraformaldehyde, and embedded in paraffin. BrdU (Roche), Ki67 (Abnova), and PCNA (DAKO) antibody staining of paraffin-embedded serial sections was performed according to the manufacturer's instructions.

Analysis of apoptosis in lung tissues

The lungs were isolated, fixed, and embedded in paraffin. The lung sections were stained for apoptotic cells using the In Situ Cell Death Detection Kit, POD (Roche), according to the manufacturer's instructions. Moreover, apoptosis was determined after counting TdT-mediated dUTP nick-end labeling (TUNEL)-positive cells using the ApoAlert® DNA Fragmentation Assay Kit (Clontech) in accordance with the manufacturer's instructions. A minimum of five different fields from each lung section was used count the number of signal-positive cells, which were represented as a percent increase or decrease.

Detection of β-galactosidase activity

Brains and lungs from the mice were freshly cut, fixed, and then stained for β-galactosidase (Cell Signaling). Briefly, slides were thawed in 37°C for 2 hours followed by fixation in 0.2% glutaraldehyde for 15 min. The slides were washed twice in 0.1 M phosphate buffer (pH 7.3) supplemented with 2 mM $MgCl_2$. The slides were then put in staining buffer containing 0.1 M phosphate buffer (pH 7.3) supplemented with 2 mM $MgCl_2$, 5 mM potassium ferrocyanide, 5 mM potassium ferricyanide, 1 mg/ml X-gal and incubated in a humidified chamber at 37°C overnight. After washing, the slides were observed under the microscope and pictures were taken.

RNA isolation and real-time quantitative PCR

Total RNA was isolated from tissues, organs, and cells using an RNeasy mini kit (Qiagen) in accordance with the manufacturer's instructions. Total RNA (1 μg) was used for cDNA synthesis with MultiScribe Reverse Transcriptase (Applied Biosystems). Quantitative PCR was performed with SYBR® Green QPCR master mix (Stratagene), and mRNA expression analyses were performed using QuantiTect Primer Assays, according to the manufacturer's instructions (Qiagen, Hilden, Germany). The primer sequences used in real-time quantitative PCR were**:**

mNLKForward	5'-AACCCTCCTCAGCACAAGC-3'
mNLKReverse**:**	5'-AGGGAATGAAAGAGCGGTGG-3'
VEGF common forward	5′-GCCAGCACATAGAGAGAATGAGC-3′
VEGF120 reverse	5′- CGGCTTGTCACATTTTTCTGG-3′
VEGF164 reverse	5′-CAAGGCTCACAGTGATTTTCTGG-3′
VEGF188 reverse	5′-AACAAGGCTCACAGTGAACGCT-3′
mGAPDHForward	5'-TCG TGG ATC TGA CGT GCC GCC-3'
mGAPDHReverse	5'-CAC CAC CCT GTT GCT GTA GCC-3'

Transient transfections and VEGF promoter luciferase assay

The luciferase activity in cells transiently transfected with reporter constructs containing deletion mutant of VEGF promoter was determined using the Dual Luciferase Assay system (Promega). The expression plasmids harboring deletion mutant of VEGF promoter were obtained from Addgene: VEGF-9kb-Luc (plasmid #27986), VEGF-5,2 kb-Luc (plasmid

#27987), and VEGF-1,6 kb-Luc (plasmid #29667). The luminescent signal was quantitated using the FLUOstar Optima plate reader (BMG Labtech). Luciferase expression was measured in cells transfected with plasmid constructs carrying the firefly luciferase reporter gene. As an internal control, plasmid pGL4.73 (Promega), containing the Renilla luciferase reporter gene, was co-transfected. The transfections were performed in Opti-MEM media containing 15 μl of Polyfect Transfection Reagent (Qiagen), and the cells were assayed for luciferase activity at 24 hours post-transfection, using reporter plasmids. Cells were transfected for 24 hours using 2 μl/ml Lipofectamine 2000 (Invitrogen) and 2 μg reporter plasmids in Optimem (Gibco) according to supplier's protocol.
